# Reducing loneliness in older adults: looking at locals and migrants in a Swiss case study

**DOI:** 10.1007/s10433-020-00577-4

**Published:** 2020-07-29

**Authors:** Annahita Ehsan, Danilo Bolano, Sylvie Guillaume-Boeckle, Dario Spini

**Affiliations:** 1 Life Course and Inequality Research Centre, Institute of Social Sciences, University of Lausanne, Quartier UNIL-Mouline, Bâtiment Géopolis, 1015 Lausanne, Switzerland; 2 Swiss National Centre for Competence in Research LIVES: Overcoming Vulnerability: Life Course Perspectives, University of Lausanne, Quartier UNIL-Mouline, Bâtiment Géopolis, 1015 Lausanne, Switzerland; 3 Unité Travail Social Communautaire, Pro Senectute Vaud, Rue de Maupas 51, 1004 Lausanne, Switzerland

**Keywords:** Older adults, Migration, Loneliness, Social capital, Intervention, Community

## Abstract

Older adults are at a high risk for loneliness, and community-based interventions can help reduce loneliness for all older adults in a community, regardless of their migration status. However, little research has investigated how older adults, including locals and migrants (in this case, internal newcomers and international expats) participate in these interventions. The “Neighbourhoods in Solidarity” (NS) are a series of community-based interventions that aim to increase social connectedness and reduce loneliness in older adults (55+) in the canton of Vaud, Switzerland. This longitudinal embedded mixed-methods study aimed to understand whether older adults (distinguishing between locals, newcomers, and expats) were aware of and participated in the NS, to assess whether participation was associated with changes in loneliness, and to identify relevant processes that could explain a reduction in loneliness. We combined a longitudinal pre/post survey (235 respondents) with ethnographic observations and informal interviews. Quantitative findings showed that individuals who participated in the NS did not have significant changes in loneliness. Qualitative findings showed that perceived migration played an important role in who participated, and that the community distinguished between two types of migrants: newcomers who spoke French fluently, and expats who did not. Individuals were only ‘local’ if they had ancestors from the town. Some newcomers and some locals used the NS as a platform to build a new sense of community within the NS, whereas expats rarely participated. This was due to linguistic and cultural determinants, institutional constraints, interpersonal relationships, and personal preferences.

## Introduction

Older adults are at an increased risk for loneliness, and loneliness among older adults is an emerging public health problem (Cacioppo and Cacioppo 2018). Older migrants are a subgroup of older adults that are susceptible to loneliness as well (Ciobanu et al. 2017), but there is little comparative research on loneliness in older locals and migrants. Furthermore, research on loneliness in older migrants often refers to international migrants (Victor et al. 2012; Fokkema and Naderi 2013; Van Tilburg and Fokkema 2020), with less attention to internal migrants. Community-based interventions can reduce loneliness in older adults by fostering social connections and a sense of belonging for all participants. While these interventions seem promising, differences in which older adults participate is unclear. This study contributes to this gap by looking at how older locals, newcomers (internal migrants), and expats (international migrants) engaged with a community-based intervention designed to reduce loneliness in older adults residing in one Swiss town, and whether participating reduced loneliness.

### Determinants of loneliness in older migrants

Loneliness refers to the unpleasant experience that occurs when a person feels that their network of relationships is deficient in some important way (Peplau and Perlman 1982). Loneliness has both emotional and social components: Emotional loneliness occurs when an individual feels a deficiency in close emotional relationships, whereas social loneliness occurs when an individual does not have a broader social network, or a sense of belonging to their environment (De Jong Gierveld and Van Tilburg 2006; Dykstra 2009; Weiss 1973). Klok et al. (2017) argues that belonging is a description of the social world (whether someone belongs to a group or not), which is different than the subjective experience of loneliness. Having a low sense of belonging within a community also predicts loneliness in older adults (Prieto-Flores et al. 2011).

The size, composition, and quality of individuals’ social networks are known determinants of loneliness (Hawkley 2015; Pinquart and Sörensen 2003). International migrants lose contact with some of their existing social networks, and are more vulnerable to loneliness (Ciobanu et al. 2017). International migrants’ ability to overcome loneliness by integrating into a new community and cultivating satisfying relationships within their host community can depend on a combination of cultural background, context, and individual characteristics (De Jong Gierveld et al. 2015). While there is less attention to internal migration and disruptions in social networks for older adults, internal migration also shifts individuals across spaces and places (White and Lindstrom 2005). Depending on the distance of migration, they could also experience a decrease in social networks and be vulnerable to loneliness.

First, cultural determinants can predict loneliness in older adults (Rokach 2019). Individuals from some cultures may have stronger expectations of social connectedness or belonging than others, and loneliness may vary based on mismatched expectations and realities (Fokkema et al. 2012). Loneliness rates for older adults vary by country (Sundström et al. 2009) and where a migrant is from. A study found that older international migrants who were from minority backgrounds in Great Britain were lonelier than locals, but that their loneliness rates were similar to their countries of origin (Victor et al. 2012). Loneliness is also related to language: In Canada, immigrants with different linguistic and cultural backgrounds were significantly lonelier than the general population, whereas immigrants with similar backgrounds were not (De Jong Gierveld et al. 2015).

Second, communities provide contexts where individuals and groups can create various forms of social capital, which refers to social resources that are embedded within social networks (Ehsan et al. 2019). Feelings of belonging to any community (regardless of whether it is their ‘own’ or a ‘host’) can predict loneliness (Rokach 2019), and being socially embedded in either a new or culturally similar community can protect migrants against loneliness (Ciobanu et al. 2017). A study in China found that older internal migrants had significantly less social capital than their local counterparts (Li et al. 2017). However, creating a sense of community belonging for international migrants may be even more complex than for internal migrants. International migrants may have multiple, sometimes complementary types of belonging: A sense of belonging to their place of settlement, to their ‘own’ group within their place of settlement, or to their place of origin. In their study on international migrants, Klok et al. (2017) suggested that having a strong sense of belonging to one’s own group is as effective against loneliness as integrating in a new group, but migrants must feel some sense of belonging (as opposed to marginalization). However, feelings of belonging to two or more places can result in *betwixt and between* identities (Grillo 2007), where migrants do not have a full sense of belonging to any place. Migrants who have lived in a new place for large proportions of their lives may be more susceptible to betwixt and between identities because they have had longer time to develop a sense of belonging to a new place.

Third, individual determinants, such as socio-economic position, professional activity, language proficiency, and health conditions, can overlap with migration to shape whether persons are vulnerable to loneliness (Ciobanu et al. 2017). Individual characteristics, such as cognition and behaviours, are important determinants of loneliness. For example, lonely individuals often believe that they have little control over their social circumstances (Cacioppo and Hawkley 2009). A scoping review on social exclusion for older adults noted that loneliness and social exclusion are cyclical, and reinforce each other (Walsh et al. 2017). Cultures and contexts influence social behaviours, and social behaviours that are normal in one context may be less accepted in another. This may contribute to unsatisfying relationships, a lack of community belonging to either their ‘own’ or ‘host’ community, and loneliness outcomes for migrants.

### Loneliness interventions for older migrants

Few interventions targeting loneliness in older adults have considered the importance of internal or international migration status. A meta-analysis identified 50 loneliness interventions, yet none focused on migrants or minority groups as the target population (Masi et al. 2011). Since then, other researchers have looked at loneliness interventions for migrant populations. A group-based loneliness intervention for older migrants who relocated to a Tokyo suburb improved loneliness and social support, though this intervention focused on individuals who had relocated within the last two years, and did not consider migrants who had lived there for longer (Saito et al. 2012). Migrants who have lived in a new place for many years may still experience loneliness, and this can vary by context. For example, individuals who migrate to small towns or villages with tight-knit communities may experience loneliness more strongly than to those moving to large cities. Smaller communities may have fewer individuals who are willing to create social connections with migrants, although this will depend on how welcoming each specific community is. Moving to a setting with existing migrant groups (who have already integrated with the host community) can help new migrants adapt to the host community more easily (Kivisto 2001). Migrants who move to settings where they cannot establish a sense of belonging to the host community will have limited opportunities to develop social relationships that protect against loneliness.

Interventions targeting social isolation in older adults tend to be more successful when they are participatory and group based (Dickens et al. 2011). A systematic review found that interventions aimed at more general audiences reduced loneliness more than those targeting lonely individuals (Dickens et al. 2011). Community-based interventions can combat loneliness for older adults by fostering social capital and a sense of belonging (Coll-Planas et al. 2017), and may benefit all residents regardless of how long they have lived in their community. While we would expect all older adults to benefit from interventions that are designed for them, research has rarely distinguished between whether older adults who are local or who have different migration backgrounds participate in these interventions.

### The Neighbourhoods in Solidarity intervention

In order to investigate whether different groups of older adults participate in community-based loneliness interventions, we collaborated with the “Neighbourhoods in Solidarity” (NS). The NS are a series of Swiss community-based participatory interventions that use an action research method to empower older adults (aged 55 and over) to actively participate and to build social capital in their neighbourhoods. By building social capital within communities, the NS hope to reduce existing loneliness for participants, prevent future loneliness, increase solidarity, and improve overall quality of life for older adults in participating communities (Zwygart et al. 2016). NS professionals create three groups that guide the intervention: A strategic group, a resource group, and a cohabitant group. The strategic group includes local partners who help with financing (political authorities), the resource group includes representatives from organizations in the area, and the cohabitant group includes residents who meet regularly to develop social connections and discuss different ideas. To date, there are over 20 different NS interventions across the canton of Vaud, Switzerland (Zwygart et al. 2016).

The NS last between three and five years, and consist of five phases: (1) Diagnostic, (2) construction, (3) project design, (4) implementation, and (5) autonomy. This study focuses on the diagnostic phase of the NS, where residents identify problems that they would like to address in their community. NS professionals send a letter to the homes of all town residents aged 55 and over, explaining the NS and inviting them to participate. They also advertise the initiative in local newspapers, flyers, and through approaching individuals on the street. This study focuses on the first stage and would reach a smaller number of participants. However, as participants had actively engaged with the NS, we would expect that they might feel less lonely after one year.

The NS project presented in this study took place in an anonymous town, in the French-speaking canton of Vaud, Switzerland. It faces Lake Léman and is surrounded by sloping vineyards and the Swiss Alps. It has a medieval town centre with cobble stone roads, buildings that date generations, a castle, and a church. There are newer residential apartments and villas constructed outside the town centre. At the time of this research, the town had around 10,000 residents, with more than a third over the age of 55. In 2018, approximately 74% of older adults in the town had Swiss citizenship (Statistique Vaud 2019). The town is near two cities that are home to multinational corporations, and had seen development in recent years. Rapid growth in the area has led to changes in infrastructure and increased cost of living.

### Aims and objectives

The overall aim of this study was to understand whether older adults (distinguishing between locals, newcomers, and expats in this study) participated in the NS, whether participation was associated with changes in loneliness, and to identify relevant processes that could explain a reduction in loneliness in the diagnostic phase of one NS case study.

More precisely, the quantitatively oriented objectives of this study were to (1) determine if different older locals and migrants (migrants refers to both newcomers and expats) living in a town with an NS had different loneliness at baseline, (2) determine whether these groups of older adults were aware of and participated in the NS, and (3) determine whether NS participants experienced a change in loneliness. The qualitatively oriented objectives of this study were to (4) understand how migration status played a role in the NS, and (5) identify potential processes that were relevant to community belonging and loneliness.^1^

## Data and methods

We used a longitudinal embedded mixed-methods design (Creswell and Clark 2007) during the diagnostic phase of one NS case study (May 2018–2019). We conducted a pre/post survey at the beginning and end of the diagnostic phase, to respond to objectives 1, 2, and 3. We used an ethnographic approach in between the two waves of the survey, to respond to objectives 4 and 5. This research was approved by the Swiss Ethics Board on research involving humans in the canton of Vaud. We obtained approval from the director of the NS and the town municipality. Participants were informed about the research. For the quantitative data collection, respondents received a letter outlining the study, and understood that returning the questionnaire by post implied their informed consent. The questionnaire was in French,^2^ and asked questions on socio-demographic characteristics, health and wellbeing (including loneliness), social capital and social participation, mobility, involvement in the NS, and more. For the qualitative part, the NS professionals and two of the authors presented the mixed-methods research project to members of the cohabitant group and resource groups on multiple occasions. Participants were aware of both qualitative and quantitative components, and that this study was a part of the first author’s doctoral research. The fieldwork took place in a public space, and no audio was recorded. The Swiss Ethics Board did not require signed consent forms for any of the research.

### Information sources and sampling

#### Quantitative data

We randomly selected a sample of older adults (aged 55+) based on civil registry data^3^ (total of 3456 older adult residents) provided by the municipality. This sample included any town resident, regardless of their migration status. The first wave of the survey was administered to 1276^4^ residents (55+) in May 2018 (*n* = 467, 37% response rate). The second wave of the questionnaire was sent to the 467 individuals who had already responded, in May 2019 (*n* = 281, 60% response rate^5^). Those who replied to both waves were on average older than those dropped-out (mean age of 70.4 vs. 68.5, t-test *p* < .05). There were no statistically significant differences in gender, health, level of loneliness, or living arrangements.

Out of 281 individuals that replied to both waves, we excluded 46 individuals with missing information on our research outcomes (loneliness,^6^ migration, knowledge of the NS, and participation in the NS). Our final analytic sample included 235 town residents. The mean age of the survey respondents was 69.4, and over half (56%) were women. On average, individuals who replied to our survey were not lonely at baseline (*M *= 1.2). The majority (71%) were born in Switzerland, and many (46%) were born in the same canton as the town. Respondent’s birthplaces are described in Table 1. More than half (54%) had spent between one and two-thirds of their lives in the town. Others had spent less than one-third (34%) or more than two-thirds (11%) of their lives in the town. While the majority of respondents (63%) had heard of the NS, relatively few (11%) had participated in it. Survey respondents who did participate in the NS were all born in Switzerland.^7^ Most of the respondents (46%) were in good health or very good health (31%). The sample was well-educated: Many respondents were university graduates (40%) or held professional diplomas from technical schools (16%). Most respondents (70%) lived with at least one other person.

**Table 1:** Respondent birthplaces

	Birth place	*n*	(%)
Switzerland	Vaud	109	46.38
	Outside Vaud	58	24.68
	Total	167	71.06
Neighbouring country	France	17	7.23
	Italy	7	2.98
	Germany	7	2.98
	Austria	1	0.43
	Total	32	13.62
Other Europe	Scandinavia	11	4.68
	Western Europe	5	2.13
	Southern Europe	1	0.43
	Eastern Europe	1	0.43
	Total	18	7.66
Outside Europe	Central Asia	1	0.43
	East Asia	1	0.43
	South-East Asia	6	2.55
	North Africa	2	0.85
	North America	4	1.70
	South America	4	1.70
	Total	18	7.66
Total		235	100.00

#### Qualitative data

Qualitative findings were based on multiple sources. The first author conducted 82 h of ethnographic observations in the context of the cohabitant and resource groups [observer as a participant stance (Gold 1958)]. The cohabitant group meetings included 50 individuals who identified and discussed any problems that they wanted to address in their community every three weeks. These sessions were open ended by design, so that the community could identify issues that are important to them. Resource group meetings had around ten stakeholders. During these monthly group meetings, representatives from associations were updated on the cohabitant group activities, shared on-going initiatives in their respective organizations, and discussed other issues concerning older adults in their town. The NS also held one community forum with around 250 participants, which provided an opportunity to conduct informal interviews and observe focus groups with individuals who did not participate in the cohabitant group. Meetings and informal interviews were not audio-recorded, as it would have been inappropriate in the setting.^8^ The first author, who was observing the group meetings, took detailed field notes, and hand wrote important quotes during meetings and informal interviews. The NS professionals provided minutes for each meeting. Finally, we analysed the *additional comments* section from both waves of the quantitative survey, to include an even larger range of perspectives in our findings. We analysed 102 comments from the first wave and 72 from the second, with 27 provided by the same respondents.

### Data analysis

#### Quantitative data analysis

##### The analytic approach

Using multivariate regression models, we examined whether groups of older adults who had been living in the town for different proportions of time had different loneliness at baseline (objective 1—linear model), whether these older adults were aware of and participated in the NS (objective 2—logit models), and if individuals who participated in the NS and replied to the questionnaire experienced a significant change in their loneliness score between waves (objective 3—linear model). We controlled for socio-demographic characteristics that could be correlated to both loneliness and participation: linear age,^9^ gender, education, whether the person lived alone or not, and self-rated health.

##### Outcome measures

Loneliness was measured using De Jong Gierveld and Van Tilburg’s (2006) six-item short scale for social and emotional loneliness. The scores ranged from 0 to 6, with 6 being the most lonely. We looked at loneliness as one construct (instead of distinguishing between social and emotional loneliness) because we wanted to keep the research in line with the NS, who view loneliness as one construct. Our qualitative fieldwork showed that there were three distinct groups: locals (who had spent their whole lives in the town), newcomers (often internal migrants within the canton who had lived in the town for decades), and expats (often international migrants who had lived in the town for less of their lives). We saw that only individuals who grew up in the town were not considered migrants. While we acknowledge that individual’s country of origin and cultural background could have also played a role in community belonging and loneliness, it was clear that both internal (migrating as little as from a neighbouring village) and international migrants saw themselves as migrants. We therefore looked at how long individuals had lived in the town for, and measured migration by how many years the respondent had lived in the town (self-reported). It would have been inappropriate to look at birthplace, since some individuals who self-identified as migrants would have been born in the same hospital as ‘locals’ in a nearby city. Since the number of years of residency might depend on the age of the respondent, we created a continuous variable representing the relative proportion of time (share of life) that all respondents had lived in the town, to account for different ages at baseline, which ranged from 56 to 95 years. We then split this into three categories: Individuals who had spent less than a third, between one and two-thirds, and more than two-thirds of their lives in the town. Finally, we measured if respondents were aware of the NS (“Have you already heard of the NS project by Pro Senectute Vaud?”, 0 = no, 1 = yes), and if they participated in the NS (“Are you involved in the NS in your neighbourhood”, 0 = no, 1 = yes). We expected respondents to be aware of the NS, since the NS had mailed pamphlets to each resident aged 55 and over a few months before the intervention started, and also sent invitations to attend a forum between both waves of data collection.

#### Qualitative data analysis

The first author coded the field notes, minutes, and questionnaire comments, then analysed them using thematic analysis (Guest et al. 2011) to address objectives 4 and 5. We translated some quotations presented in the text to English for coherence and further anonymization.

## Results

### Perceived migration status

Town residents perceived different *types* of migrants. NS participants repeatedly mentioned that there were three distinct groups in the town: Locals, newcomers, and expats. Newcomers and expats were both considered migrants. Interestingly, whether or not individuals were perceived as migrants was not related to their country of origin or their nationality, but rather a mix of how long they had resided in the town and whether they were francophone.

#### Locals: “I’m losing my town”

Locals, also referred to as “real villagers” by francophone cohabitant group members and other town residents in questionnaires, grew up in and usually had ancestors^10^ from the town. Local families were generally well-known and had a role in the town’s history. Many locals felt a strong sense of belonging and ownership of the town, and some saw recent changes in the environmental and socio-cultural landscape as threats to traditional ways of life. These changes were sometimes accompanied by resentment towards migrants who disrespected social norms:

Before, [the town] was a pretty village with vineyards, where we could find local shops with essential goods. Now, these old shops have disappeared, and we are left with unknown offices, marketing platforms, and housing agencies where people speak English. We don’t feel at home anymore, there’s no one but Anglophones, Russians, Asians, and strangers, who think they are entitled to everything just because they’ve bought a property in our village. I like to walk on the quay in the morning, where we can see real villagers. In short, we’ve been invaded!—Woman in her fifties, written in questionnaire.

Most of the resentment felt by locals was towards wealthy foreign-born migrants who did not speak French. Migrants who were more similar in language and culture were perceived as less threatening.

#### Newcomers: “It’s the locals versus the new inhabitants”

Newcomers were individuals who had moved to the town from a Swiss, and often French-speaking area. Newcomers viewed themselves as migrants, even if they had moved small distances within Switzerland, and had lived in the town for decades. One woman who had moved from a neighbouring town introduced herself by saying:

I’ve been living here for 20 years. I’m an immigrant.—Woman in her seventies, during cohabitant group meeting.

Newcomers seemed aware of what it meant to be local, and many felt that they did not belong to this group, even if they were from nearby towns with similar histories and cultures. During an informal interview, one man referred to locals as “the clan in the town centre”, emphasizing the impermeableness of the group. This perceived lack of belonging or acceptance contributed to newcomers feeling like migrants, regardless of moving very short distances.

#### Expats: “I’ll never be truly included because I speak French with an accent”

Both locals and newcomers considered expats to be wealthy individuals who were not fluent French speakers. They perceived expats to have minimal appreciation and respect for the area, and to be generally unwilling to adapt to new ways of life and participate in town organizations or activities. Some residents viewed expats as individuals who were increasing the cost of living in the town. An issue that came up during cohabitant group meetings and in questionnaires was a fear that future generations could no longer live there:

There are already too many of us! We need to stop attracting rich people so that our descendants will be able to keep their patrimonies!—Man in his seventies, written in questionnaire.

Some expats were aware of how others saw them. During informal interviews, two women disclosed that they had received negative comments after purchasing homes in the area, and that they made locals feel uncomfortable. Other expats seemed less aware of the town’s history and culture:

I’ve been living here for almost 20 years and can’t imagine myself anywhere else… but right now, it feels like a bedroom community^11^ serving [city] and [city].—Woman in her sixties, during informal interview.

Varying perceptions of the town may have had to do with differences in social connections, feelings of belonging, and socio-cultural understanding of their surroundings. Expats often came from larger cities, and may not have understood norms that seemed obvious to locals. This could have resulted in behaviours that came across as impolite, such as not greeting neighbours or addressing others informally.

### Migration status and loneliness

Time spent in the town was significantly associated with loneliness at baseline, even after controlling for demographic variables. Individuals who lived in the town between one and two-thirds (*B* = − .496 (95% CI [− .95, − .04], *p* < .05) and for more than two-thirds (*B* = − .758 (95% CI [− 1.39, − .12], *p* < .05) were significantly less lonely than individuals who had spent less than one-third of their lives in the town (Table 2). Those with a better level of health reported also lower levels of loneliness (*B* = − 1.420 (95% CI [− 1.91, − .93], *p* < .01).

**Table 2:** Loneliness score at baseline: linear model

Variables	Loneliness at baseline
*Time spent in the town (ref. less than 1/3 of life time)*	
From 1/3 to 2/3 of life time	− 0.496** (0.231)
More than 2/3 of life time	− 0.758** (0.323)
*Demographics*	
Female	0.236 (0.214)
Age	0.001 (0.013)
University degree	0.058 (0.218)
Living alone	0.380 (0.232)
Good level of health	− 1.420*** (0.249)
Constant	2.301** (1.006)
Number of respondents	235
R-squared	0.212

Standard errors in parentheses

***p < 0.01; **p < 0.05; *p < 0.1

### Migration status and participation in the NS

Individuals who spent less time living in the town were less aware of the NS (Table 3—first column), even after controlling for demographic variables and loneliness at baseline. Individuals who had spent more than two-thirds of their lives in the town were on average three times more aware of the NS than those who had lived there for less than a third of their lives (OR 3.062, *p *<.05), and individuals who had spent between one and two-thirds of their lives in the town were also more aware of NS (OR 1.688, *p *< .01).^12^ Similar results were observed for the association between how long individuals lived in the town and if they participated in the NS (Table 3—second column). The magnitude of this effect was similar as for the association between how long individuals lived in the town and if they were aware of the NS, but these effects were not statistically significant, probably due to sample size. Women and older respondents were more likely of both being aware of and participating in NS.

**Table 3:** Knowledge (first column) and Participation (second column) in NS intervention: logit model: odds ratios

Variables	Aware of NS	Participated in NS
*Time spent in the town (ref. less than 1/3 of life time)*		
From 1/3 to 2/3 of life time	1.688* (0.531)	1.644 (0.892)
More than 2/3 of life time	3.062** (1.618)	3.016 (2.143)
Loneliness score at baseline	1.048 (0.097)	0.957 (0.144)
*Demographics*		
Female	1.927** (0.593)	2.312* (1.157)
Age	1.053*** (0.020)	1.077** (0.031)
University degree	1.583 (0.483)	1.806 (0.885)
Living alone	0.867 (0.285)	1.479 (0.723)
Good level of health	1.130 (0.418)	0.666 (0.358)
Constant	0.015*** (0.024)	0.000*** (0.000)
Number of respondents	235	235

Standard errors in parentheses

***p < 0.01; **p < 0.05; *p < 0.1

### Participation in the NS: the unexpected role of perceived migration status

Most individuals who participated in the NS were French-speaking newcomers, although some locals participated as well. Newcomers often viewed locals as a closed, impermeable group who could be identified by their last names. While locals seemed like a cohesive group to outsiders, not all locals felt this way: One man shared that he no longer felt like part of the local group after he moved to the residential area outside the town centre. For him, moving less than a kilometre had changed his sense of belonging, and he was seeking new connections within the NS.

Participants often mentioned that they were participating in the NS to get to know their neighbours, and to develop a new community with stronger social connections. They did not specify whether the new community they desired distinguished between locals, newcomers, or expats, although this may have been because the fieldworker was an expat herself. What many newcomers did say, was that they needed the NS to help build community because they felt excluded by locals, and had difficulties communicating with expats. Members of the cohabitant group regularly mentioned language as an important barrier to their sense of cohesion and community, as well as a difficulty in getting to know expat neighbours:

There are places where we can’t even get to know our neighbours because they don’t speak the same language as us.—Woman in her eighties, during cohabitant group meeting.

Cultural barriers were also discussed in the NS. There was a large consensus that expats were not adequately integrated, and that the NS should target this. During the first cohabitant group meeting, one man referred to this as “resolving the Anglophone problem”, and another proposed a solution:

We need to help the Anglophones assimilate because they don’t associate with anyone.—Man in his seventies, during cohabitant group meeting.

The idea that expats did not generally wish to participate in local activities or organizations was a recurring theme. This was partially true: Many expats had existing social networks outside of the country, and did not wish to participate in community activities. Expats who felt lonely and could have benefited from participating may have developed other coping mechanisms:

Of course, I felt really lonely for the first two years. But I’ve learned not to have expectations, and that’s helped me a lot.—Woman in her sixties, during informal interview.

Expats could also participate in other initiatives within the town, such as an English-speaking international women’s group. Expats may have chosen to attend community activities outside of the NS, where they felt more accepted and could communicate more easily.

### Processes that led to participation in the NS

Perceived migration played a role in the diagnostic phase of the NS. Both cohabitant and resource groups questioned the target population, and whether it was possible to include locals, newcomers, and expats in one group. Members of the cohabitant group regularly discussed whether the NS should dedicate resources to include expats, or whether they should foster a sense of community and solidarity for individuals who were already willing to participate. It is important to note that the NS professionals and many of the cohabitant group members did not actively exclude anyone from participating. One of the NS professionals had focused her diagnostic interviews^13^ on migrants in order to include them in the process. Some members of the cohabitant group were pro-integration, had lived abroad themselves, and proposed solutions to include expats.^14^ However, the perceived group atmosphere may have dissuaded some expats from participating.

Various expats came to one or two cohabitant group meetings but did not return. They expressed they did not participate in the NS because it was not *meant* for them, but for French speakers. This came across in questionnaires and informal interviews. They may have felt this way because of linguistic barriers, as well as interpersonal challenges they faced when they tried to join the group. For instance, micro-aggressive language such as “assimilate”, and the “Anglophone problem” had offended expats who tried to participate in cohabitant meetings. One woman (who spoke French but was from the United States) attended the first cohabitant group meeting because she wanted to help include non-French-speaking expats in the community. After the meeting, she told the fieldworker that she did not feel like she was taken seriously, and that she would not waste her time by returning.

Qualitative data revealed that very few expats participated in the NS, but those who did spoke French at a level that was advanced enough to contribute to discussions. Despite this, strong accents got in the way of whether or not they felt included. During an informal interview, a woman said that other members of the cohabitant group would speak to her during the NS, but refused to speak to her when they saw each other in the town. She thought it was because she lived in the town centre and spoke French with an accent. Another woman who came to a couple cohabitant group meetings told the cohabitant group that they would have to tolerate her accent. Her behaviour showed that she had anticipated a negative reaction, and reflects what many other expats felt: Other town residents were hesitant to accept them.

#### Institutional barriers to migrant participation in the NS

The majority of NS participants were locals or newcomers, and this could be partly due to the NS process, which was designed for the entire population and did not keep barriers to expat participation in mind. The NS professionals were Francophone, approached individuals in the street in French, and sent French flyers by mail. After some individuals showed interest, they used these participants and snowballing as a technique to recruit more participants. This strategy utilized existing social networks, and participants often recruited existing acquaintances. In the questionnaires, one person wrote that it was difficult for them to participate in the NS because everyone already knew each other, and that the NS appeared to be a clique rather than a program for those who are truly alone.

Institutional and municipal constraints also led to barriers to participation. The NS professionals were assigned a larger geographic area than usual (entire town instead of a neighbourhood), without additional resources to contact more individuals, and to hold more meetings at various times. Meetings always took place during the day, and one person wrote that it was impossible for working individuals to participate. Furthermore, some individuals in the resource group had strong opinions about expats. For example, a municipal representative told the first author that she could not distribute questionnaires in English, because the NS was for individuals who wanted to integrate as opposed to those who did not speak French. Barriers at these higher levels interacted with who was approached and who participated.

### Participation in the NS and loneliness

Only 25 participants in the NS responded to both waves of the questionnaires, and there was not enough power to obtain statistically significant results. Individuals who participated in the NS and who replied to the questionnaire were all Swiss-born. These persons reported a slightly higher reduction in loneliness than those who did not (average of − .4 points on the loneliness scale as opposed to − .13),^15^ but this difference was not statistically significant. Migration status was not statistically associated with changes in loneliness scores among those participated in the program. These results are reported in Table 4.

**Table 4:** Change in loneliness score between waves: linear model

Variables	
*Time spent in the town (ref. less than 1/3 of life time)*	
From 1/3 to 2/3 of life time	0.187 (0.207)
More than 2/3 of life time	0.352 (0.315)
*Demographics*	
Gender	− 0.019 (0.198)
Age	0.022* (0.012)
University degree	0.154 (0.198)
Living alone	0.209 (0.213)
Good level of health	0.288 (0.224)
Constant	− 2.236** (0.952)
Number of respondents	235
R-squared	0.032

Standard errors in parentheses

***p < 0.01; **p < 0.05; *p < 0.1

## Discussion

Quantitative findings showed that older adults who lived in the town for less than a third of their lives were lonelier than other town residents, and residents who spent over two-thirds of their lives in the town were the least lonely (objective 1). Individuals who lived in the town for longer were more aware of the NS, but there was no statistically significant difference in whether individuals participated in the NS or not (objective 2). The quantitative sample only had Swiss-born NS participants: No NS participants who were expats replied to our questionnaire. Therefore, even though there was no statistical difference in participation between individuals who had lived in the town for different lengths of time, we cannot differentiate between locals, newcomers, and expats with this data. Finally, participating in the diagnostic phase of the NS was not significantly associated with changes in loneliness scores (objective 3).

Qualitative findings showed that there were three distinct groups: Locals, newcomers, and expats. The latter two groups were both considered ‘migrants’, but in different ways: Newcomers who had similar language and culture to locals, and expats who were viewed as very different to locals. Newcomers saw themselves as migrants despite moving short distances and living in the community for decades. These divisions were prominent in cohabitant group discussions, and subsequently played a role in who was included in the NS (objective 4). Overall, the distinction between locals, newcomers, and expats affected group dynamics and the NS process, which led to some expats feeling less included in the NS (objective 5).

### Barriers to participation in community-based loneliness interventions

Our case study showed that older adults’ participation in the NS was based on a complex interaction between language and culture, institutional constraints, interpersonal relationships, and personal preferences.

First, language and culture played a role in how migrants were perceived. Expats often came from different cultures, and international migrants who do not speak the local language can have difficulties integrating with the host community (even if they may have their own communities) (De Jong Gierveld et al. 2015). Newcomers with similar language and culture were also identified as migrants, despite being from the same country or area, and living in the town for multiple decades. Identifying as a migrant played a role in individuals’ sense of belonging to the town, and often served as motivation for newcomers to participate in the NS.

Second, institutional constraints played a role in why locals and newcomers were more present at the NS than expats. Locals and newcomers both spoke French. The diagnostic phase of the NS was not designed to actively include persons who were uncomfortable speaking French (often expats), and municipal representatives initially wanted to keep the project for French speakers only. This was critical: By diffusing information in one language, expats who did not have French skills were less likely to participate to begin with. Interestingly, expats who did have French skills but who spoke with an accent also felt unwelcome. These institutional barriers limited expat knowledge and access to the NS, and could have excluded expats who may have liked to participate in the NS. This exclusion could lead to a lack of belonging, and increase loneliness within individuals who want to participate but who are excluded. Unfortunately, as the NS is designed for the community as a whole (and not specific subgroups), these barriers to participation were only identified through this research.

Third, interpersonal interactions within the NS could have affected expats’ willingness to return. Certain behaviours can be perceived as impolite in one culture and not another (e.g., addressing someone informally). The NS provided a platform for individuals to come together, but did not address social behaviours (e.g., making fun of accents) that discouraged individuals from returning. Community-based interventions should ensure that interpersonal interactions are always conducted in a welcoming and respectful environment.

Finally, personal preferences can play a role in whether or not individuals choose to participate in their communities. Migrants from different cultural backgrounds cope with loneliness in different ways (Rokach et al. 2004), and some migrants may prefer to maintain existing high quality relationships outside of their town, or develop relationships with culturally similar individuals. In this case study, expats may have chosen to foster connections in the closest city, where there were more individuals with similar backgrounds. Furthermore, multiple senses of belonging are related to loneliness (Klok et al. 2017), and certain migrants may choose *not* to develop a second or third sense of belonging to a community. However, the choice to foster connections in the town (or not) could also be related to feeling welcome (or unwelcome) to begin with.

### Recommendations for community-based loneliness interventions

This case study was conducted during the diagnostic phase of the NS, which as part of the action research process, aims to identify what older adults view as problems they would like to address within their town. Group meetings were discussion-heavy and attracted a smaller group of individuals who already feel strongly about their communities. More individuals may choose to participate in the next phases of the NS, where activities will attract a broader audience.

Many of the barriers to participation described in this case study were neither intentional nor anticipated by the NS professionals. Community-based interventions depend highly on contexts and group dynamics within these contexts. Interventions should dedicate resources to understanding communities (such as the diagnostic phase of the NS), in order to develop contextually appropriate interventions that include different groups. Special attention should be given to who is encouraged to participate.

Community-based interventions implicitly target population subgroups, and this should be explicitly considered in the design. Not all older adults want to join community activities (Curry and Fisher 2013), but those who do should have equal opportunity if the intervention is designed for the community as a whole. Sense of belonging is a better predictor of immigrant loneliness than participation in local associations or supportive relationships with neighbours (De Jong Gierveld et al. 2015), and loneliness interventions should strive to foster inclusive communities where all individuals can develop a sense of belonging if they choose. They should understand how different types of migrants and locals see one another, and establish contextually relevant strategies that address language and culture, institutional constraints, interpersonal relationships, and personal preferences.

### Strengths and limitations

The embedded mixed-methods design used in this study provided complementary perspectives on how migration played a role in if, why, and how older adults participated in a loneliness intervention for older adults. This resulted in a deeper, contextualised understanding of town dynamics and challenges that were faced by different groups in this case study. Finally, this research directly informed the next phases of this case study: After findings from this study were disseminated, NS professionals took special measures to be more inclusive to expats, such as offering activities in English.

Despite the strengths, we only looked at the diagnostic phase of the NS, which targets a small group of individuals by design. Around 40 individuals regularly participated in cohabitant group meetings, and 25 responded to both waves of our survey. All 25 participants who responded were Swiss-born, and we were unable to observe statistically significant changes in loneliness due to our sample size. The questionnaires were distributed in French, which limited responses from non-French speakers in our quantitative sample (selection bias).

Finally, qualitative field notes were written and analysed by a bilingual Canadian expat residing in Switzerland. This may have influenced her observations, how she understood interactions, and what participants were willing to disclose to her. She tried to overcome this bias by analysing the *additional comments* section in the pre/post survey. She also had regular discussions with the third author, who is Swiss-born and facilitated all group meetings.

## Conclusion

Perceived migration is complex and related to feelings of belonging to a community. Older adults may view themselves as migrants, even after moving short distances in the same region. Migrants with similar socio-cultural norms, such as speaking the same language, may be able to participate in community-based interventions more easily than migrants with different socio-cultural backgrounds. Future community-based interventions targeting loneliness in older adults should consider whether migrants participate, what it means to be a migrant, how different groups perceive migration, and how these factors interact with other social structures to shape community dynamics. They should also consider potential barriers in terms of language and culture, institutional constraints, interpersonal relationships, and personal preferences. Overall, this research shows that perceived migration can play a role in whether older adults participate in community-based interventions to reduce loneliness.

## Footnotes

1 The qualitatively oriented objectives were intentionally vague as we were using an ethnographic approach designed to see what emerged from the field (O'Reilly 2012).

2 The municipality did not give us permission to distribute it in other languages.

3 Swiss civil registries are regularly updated and include every individual who resides in the municipality.

4 The sample in the first wave was obtained by randomly selecting 1200 inhabitants from the civil registry. We also sent questionnaires to 76 individuals who had attended an information session and shared their contact information with the NS. This strategy ensured that potential NS participants were included in the quantitative sample.

5 We are unsure why the attrition was so high. It could have had to do with deaths, individuals moving, or a lack of interest as they had already replied to one of the questionnaires just one year before. We did not provide any form of monetary or nonmonetary incentives to reply to the survey, and we did have re-contacting strategies for drops-out. This might have influenced the overall non-response rate.

6 We did not score loneliness if at least one item of the De Jong Gierveld and Van Tilburg short scale was missing (De Jong Gierveld et al. 2012). Those with a lower level of health and older respondents were more likely to have at least one missing item of the loneliness scale. Migration status, gender, level of education, living arrangement were not statically associated with increasing chances of not responding.

7 However, qualitative data revealed that at least three participants were born outside of Switzerland but had resided in the town for decades.

8 Group meetings usually consisted of around 40 attendees, and getting consent for all of them to be audio-recorded prior to each meeting would not have been feasible. The NS professionals were uncomfortable with audio recordings of group meetings, and suggested that audio-recording individuals during informal conversations would have made them less likely to disclose information concerning group dynamics or relationships with other individuals in the group. We therefore chose to take detailed notes during meetings, and wrote quotes that stood out by hand in quotation marks.

9 We also tested the model controlling for quadratic age, but it did not change the results.

10 Translated from *famille de souche*, which roughly translates to English as families with roots in the area.

11 Bedroom communities, also known as commuter towns, refer to towns that are primarily residential. Individuals who live in these communities tend to work in nearby cities. The general connotation is that residents only sleep in the town.

12 We have not reported confidence intervals for the odds ratios, as they are not symmetrical due to a transformation from the linear model.

13 Interviews that NS professionals conducted with residents before the cohabitant group began.

14 Solutions proposed were on including young expat families who had recently migrated to the area, in order to help them and their children integrate more easily. These solutions did not extend to older expats.

15 The change of loneliness score in our sample ranged from − 4 to + 4.
